# Distinguishing crystallization stages and their influence on quantum efficiency during perovskite solar cell formation in real-time

**DOI:** 10.1038/s41598-017-13855-6

**Published:** 2017-11-02

**Authors:** Lukas Wagner, Laura E. Mundt, Gayathri Mathiazhagan, Markus Mundus, Martin C. Schubert, Simone Mastroianni, Uli Würfel, Andreas Hinsch, Stefan W. Glunz

**Affiliations:** 1 Fraunhofer Institute for Solar Energy Systems ISE, Heidenhofstraße 2, D-79110 Freiburg, Germany; 2 Freiburg Materials Research Center FMF, Albert-Ludwigs-University Freiburg, Stefan-Meier-Straße 25, D-79140 Freiburg, Germany; 3 Laboratory for Photovoltaic Energy Conversion, Albert-Ludwigs-University Freiburg, Emmy-Noether-Straße 2, D-79110 Freiburg, Germany

## Abstract

Relating crystallization of the absorber layer in a perovskite solar cell (PSC) to the device performance is a key challenge for the process development and in-depth understanding of these types of high efficient solar cells. A novel approach that enables real-time photo-physical and electrical characterization using a graphite-based PSC is introduced in this work. In our graphite-based PSC, the device architecture of porous monolithic contact layers creates the possibility to perform photovoltaic measurements while the perovskite crystallizes within this scaffold. The kinetics of crystallization in a solution based 2-step formation process has been analyzed by real-time measurement of the external photon to electron quantum efficiency as well as the photoluminescence emission spectra of the solar cell. With this method it was in particular possible to identify a previously overlooked crystallization stage during the formation of the perovskite absorber layer. This stage has significant influence on the development of the photocurrent, which is attributed to the formation of electrical pathways between the electron and hole contact, enabling efficient charge carrier extraction. We observe that in contrast to previously suggested models, the perovskite layer formation is indeed not complete with the end of crystal growth.

## Introduction

In the past few years, great improvements of performance and stability of hybrid organic-inorganic perovskite solar cells (PSCs) have been achieved by solution processing^1–5^. Tailoring the contact materials and absorber material composition^4,5^ as well as optimizing the perovskite formation procedures^6,7^ enabled conversion efficiencies above 20%^8^.

In order to control the formation of perovskite layers in high-efficiency solar cells, understanding the crystallization kinetics and its influence on the photovoltaic performance is fundamental. So far crystallization kinetics of perovskites have only been studied extensively on free-standing films^9–16^. To fabricate complete cells, after perovskite film formation, a consecutive deposition of the counter electrode is needed for charge extraction. Therefore, with previous approaches it was not possible to directly relate the perovskite cell performance to the crystallization kinetics and a method to monitor the photo-physical and electric properties from the very beginning was still pending.

**Figure 1:**
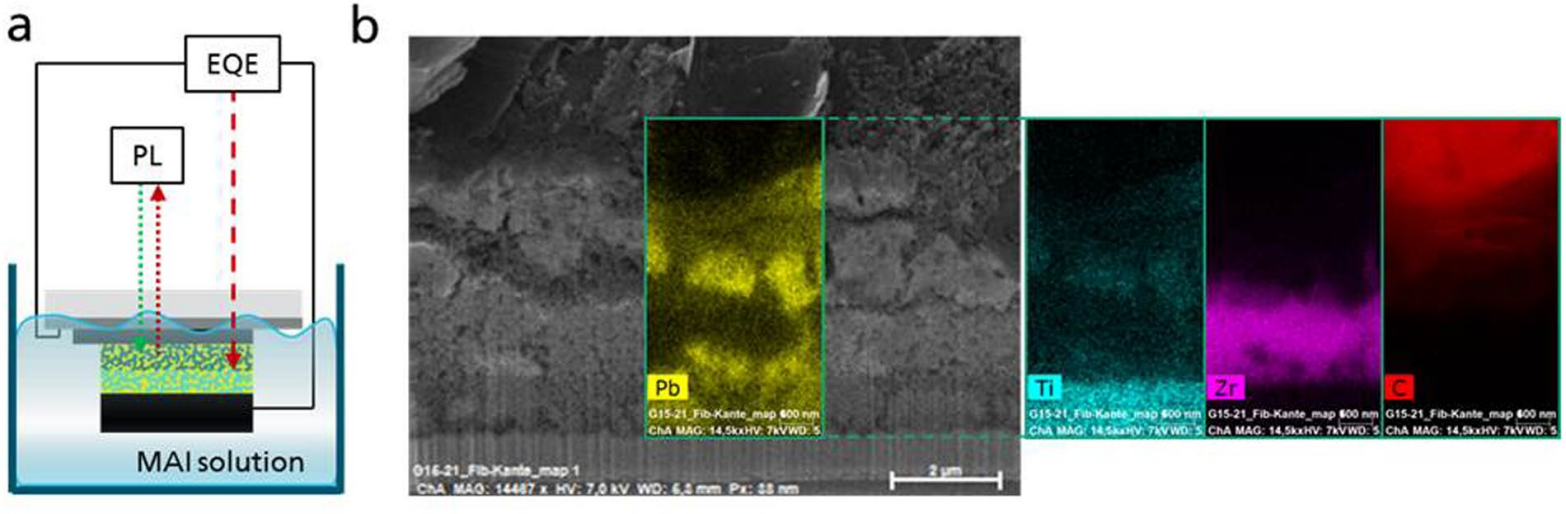
Experimental setup for real-time crystallization monitoring. (**a**) Illustration of the experimental setup for real-time PL and EQE monitoring during perovskite formation upon immersion in MAI solution. (**b**) SEM image along the cross-section a perovskite filled graphite cell and close-up images of EDX-maps for the elements Pb (indicating perovskite infiltration inside the porous scaffold), Ti (indicating the porous TiO_2_ electron selective layer), Zr (indicating the porous ZrO space layer) and C (indicating the porous graphite back electrode).

In this work, we present an approach to directly link the photovoltaic evolvement and the crystallization in real-time throughout the formation of the perovskite solar cell. We performed real-time characterization, showing the external quantum efficiency (EQE) at fixed wavelengths as well as the spectrally resolved photoluminescence (PL) emission throughout the entire process of the perovskite crystal formation (cf. Fig. 1a). Direct charge extraction during crystallization is enabled by using a monolithic contact scaffold with a graphite counter electrode^17–20^. We form the perovskite by a standard 2-step reaction process^21^. Thereby, the inorganic metal-halide salt (most commonly lead iodide, PbI_2_) is first applied and interacts in a second step with a metal-organic halide salt (most commonly methyl ammonium iodide, MAI), dissolved in a non-polar solvent.

After an exhaustive STN literature search, we come to the conclusion that this is the first time that the photovoltaic effect of a solid state solar cell device is observed during crystal formation. In the following, we demonstrate the potential of this technique to link the photovoltaic properties to the critical kinetics of perovskite conversion and crystallization from solution.

## Results

### Photoluminescence measurements

**Figure 2:**
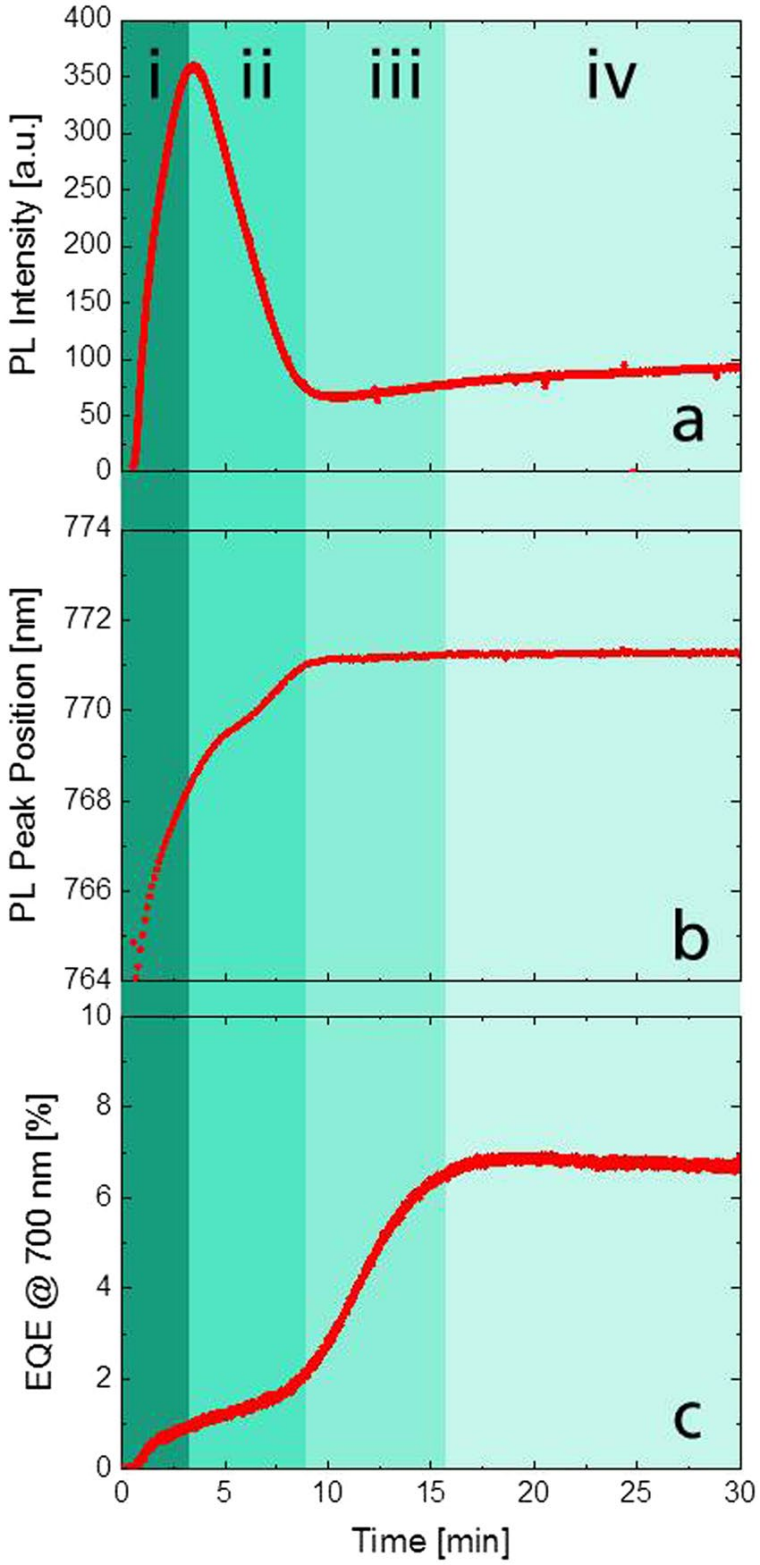
Real-time measurements of photo-physical and electrical properties during perovskite crystallization. (**a**) Photoluminescence intensity and (**b**) spectral peak position as well as (**c**) EQE measurements performed at 700 nm throughout the four reaction stages (i to iv) during MAI-immersion.

For the formation of the perovskite, the PbI_2_-filled porous cell structure is immersed in MAI solution. The formation of perovskite can be observed by the naked eye as the yellow PbI_2_-film turns dark within 9 minutes (cf. Supplementary Fig. S1). Deeper insight in this reaction can be gained from the photoluminescence (PL) signal of the perovskite. Figure 2 shows the PL intensity (Fig. 2a) and the spectral position of the PL peak (Fig. 2b) during this reaction. These parameters were obtained from a Gaussian peak fit to the spectrally resolved PL data. From the change of dynamics in the PL signal we can directly distinguish three different stages during perovskite crystallization, as indicated by different coloring in Fig. 2: A stage i) in which a strong PL intensity rise peaks after approximately 3 min of MAI dipping. And a second stage ii) in which the signal decreases until minute 8. After this point the signal sharply turns into steady state. The occurrence of the same stages is also observed in the behavior of the spectral peak position which exhibits a red-shift throughout the first 8 minutes before reaching a stable level at 771 nm. The spatial distribution of the PL emission of the complete cell as measured with a confocal PL microscope (cf. Supplementary Fig. S5) reveals a homogeneous PL throughout the active area of the analyzed cell.

### Photovoltaic effect

The external photon to electron quantum efficiency (EQE) at 700 nm was traced as displayed in Fig. 2c. The EQE rises throughout stages i), ii) and iii) and after 15 minutes remains at an almost stable value, marking the onset of stage iv). Revealingly, in the first three stages the EQE increases in distinct rates: there is a strong but short rise in stage i), followed by a slower rise in stage ii). The most significant increase in EQE takes place in stage iii). We note that the onsets of the stages do not match completely between the PL and the EQE at 700 nm as they were recorded on different cells. Illumination with 700 nm wavelength was chosen from the flat region of the EQE spectral curve, which significantly contributes to the overall short-circuit current. Also this wavelength is suitable in order to avoid current contributions from possible electrochemical mechanisms given by the light absorption of TiO_2_ and PbI_2_ in the presence of an electrolyte-like solution (MAI in 2-propanol), as discussed in the Supplementary Information.

## Discussion

**Figure 3:**
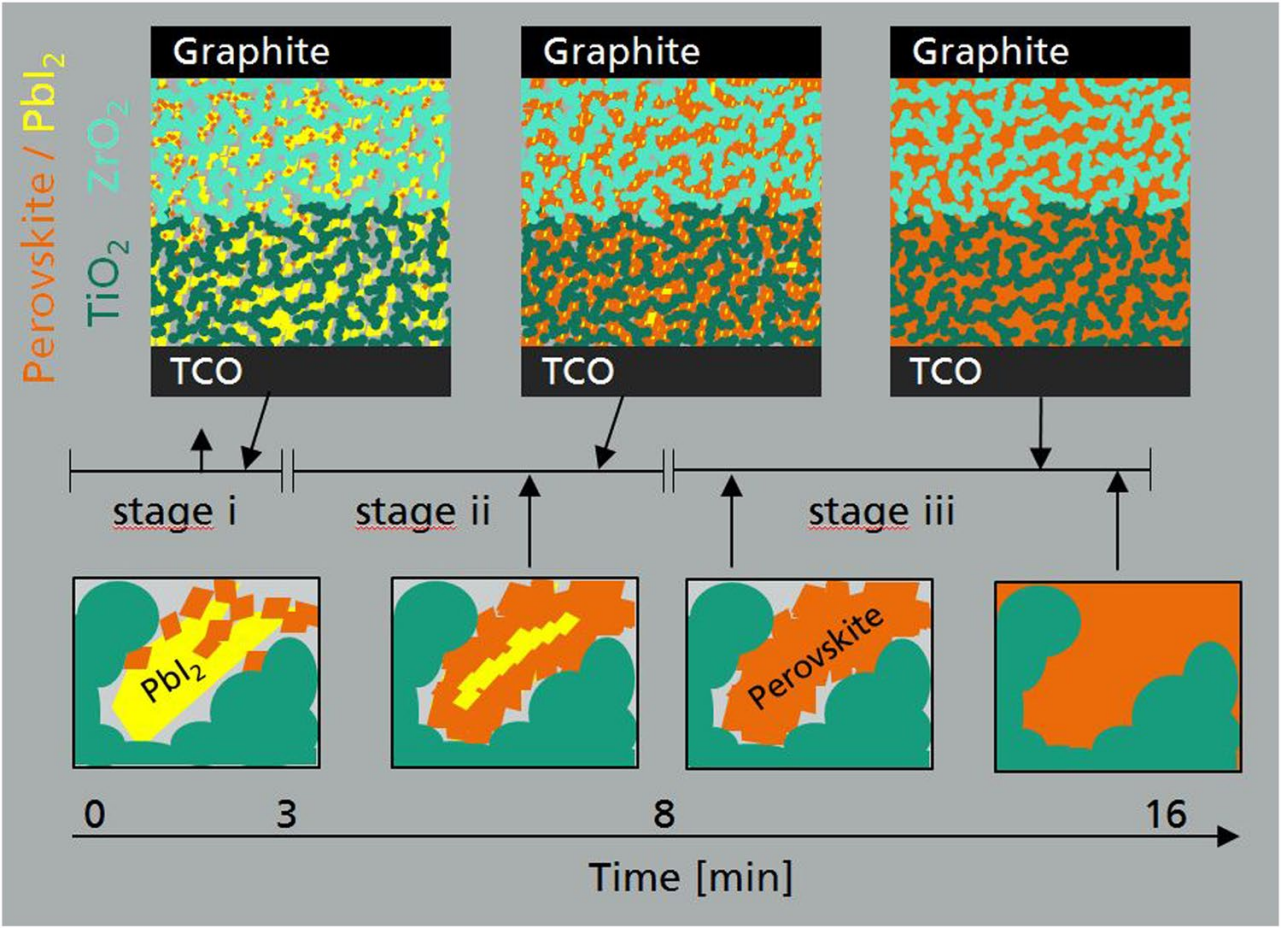
Schematic evolution of 2-step perovskite formation during MAI-immersion. Displayed are the first three stages of internal perovskite formation for the entire solar cell stack (top) and a magnified view on a single pore (bottom): After a nucleation of single perovskite crystals on the PbI_2_ surface (i) the entire PbI_2_ grain is converted gradually from the shell to the center until the conversion to perovskite is complete (ii). Finally, the perovskite reorients inside the pore for an optimal embeddedness (iii).

From the above findings that the PL intensity is peaking already in the earlier stage of the perovskite growth while the mayor part of the photocurrent develops significantly later, we suggest the following model for the 2-step perovskite reaction process from solution (cf. Figure 3 for a schematic illustration):

In stage i), the initial reaction with the MAI solution results in perovskite nucleation on the outer shell of the PbI_2_ grains^10,14,15,22,23^ as has already been suggested by Liao *et al*., for planar devices^24^. The increase of perovskite amount in this initial stage is reflected back in a rise of the PL intensity (cf. Fig. 2a).

Looking at the spectral position of the PL peak enables to relate the observed increase in PL intensity also to the growth of the crystals. The spectral position of the PL peak provides information about the band gap of the perovskite absorber. Previous investigations of the correlation of crystal size and spectral position of the perovskite PL peak revealed that small crystals exhibit a wider band gap than larger crystals^25–29^. Based on this observation, the interpretation of the time-dependent red shift of the spectral peak position as shown in Fig. 2b until the end of stage ii) can therefore be attributed to the growth of individual perovskite crystals, which is in good agreement with previous observations for the 2-step process^14,16^. Competing effects from spectral reabsorption is assumed to not play a major role in this trend since this would result in a reduced PL peak width, which is not observed (cf. Supplementary Fig. S4).

In stage i), the onset of the photocurrent, as reflected from the EQE at 700 nm, initially remains at an overall low level. The presence of a photocurrent requires that generated charge carriers are extracted from the contact layers. At this stage this means that not all crystals are electrically isolated but there are already to some extent perovskite crystals forming electrical pathways between the TiO_2_ and the graphite contact layers. As discussed in the Supplementary Information, we note that also an additional charge transport operated by the MAI-solution might contribute at a low fraction to the EQE.

In the evolution of the PL peak position and EQE in stages i) and ii) we observe a clear distinction of kinetics. We attribute this behavior to a fast surface reaction between the MAI-solution and the pristine PbI_2_ crystals, followed by a diffusion limited transport of MAI through the resulting perovskite shell, in accordance to the findings of Miyadera *et al*.^22^. The most distinct behavior in stage ii) is the strong decrease of the PL intensity which we interpret as a result of the merging of individual crystals which favors non-radiative energy transfer. At the end of stage ii), the photoactive layer is completely filled with perovskite which is also evident from the optically dark appearance of the device (cf. Supplementary Fig. S1). Comparable peaking of the photoluminescence and correlations to data from XRD have already been observed in literature^13^ for crystallization of bare perovskite films fabricated by a 1-step process^30,31^ where the perovskite crystals are formed from a single solution comprising the perovskite precursor materials. It was presumed that, as no change in the PL-signal is observed, also the perovskite formation should be completed. In our work however, employing the real-time monitoring of the EQE it seems that this presumption is incomplete. Our results indicate the existence of an additional third stage during which the main increase in the EQE is observed as shown in Fig. 2. Therefore, it is evident, that although the conversion from PbI_2_ to perovskite is mostly completed and further growth of perovskite has ended, there is still a “maturation” process going on which enables efficient charge transport and extraction.

A possible explanation for this behavior could be the reorientation of the perovskite on the surface of the porous charge extraction scaffolds, facilitated by the presence of mobile ions in the solution. Such an effect of perovskite reorientation has been reported in a different context by Hsieh *et al*. in SEM investigations of 2-step formation of perovskite on porous TiO_2_ layers^16^. In any case, stage iii) leads to the establishment of improved electrical pathways between the front and back contact as indicated by the strong increase of EQE at 700 nm. Finally, in stage iv) both the photoluminescence and the EQE signals are stationary which shows that the solution based 2-step formation process is completed.

In conclusion, understanding the relations between crystal formation and photovoltaic performance of perovskite solar cells is a major challenge for an in-depth understanding and the development of optimal fabrication procedures of these novel devices. In the literature reported so far, measurement of the photovoltaic properties of perovskite solar cells and of solid state solar cells in general has only been possible at the end of the fabrication process. Here, we introduced a novel time-resolved real-time probing of photophysical and electrical solar cell parameters during perovskite crystal formation in complete graphite-based monolithic perovskite solar cells. The capabilities of the method was demonstrated exploring the crystallization mechanisms of a 2-step solution based perovskite reaction process. Photoluminescence (PL) measurements enabled real-time monitoring of the perovskite crystal formation. A sharp initial PL intensity increase and the red-shift of the PL peak position reflected an increase in the crystal size during perovskite conversion within the scaffold pores. In a consecutive stage, the PL intensity decreases and a further red-shift of the peak position indicated a further growth of the perovskite grains to a point where boundary effects further quench the PL intensity. A stable grain size was observed by the end of this second stage. Surprisingly, although the crystals did not alter further in size, the photocurrent of the cell, as reflected by real-time measurements of the EQE, still remained marginal. The most significant increase in photocurrent was observed after this point, which we ascribed to improved electrical connection to the contact layers by a final allignment of the perovskite surface to the porous scaffold. The identification of this third stage subsequent to the finalization of crystal growth highlights the potential of this novel real-time method. The possibility to access the photovoltaic properties of the evolving perovskite layer can therefore be used to refine crystallization models and to gain better control on fabrication processes.

## Methods

### Perovskite solar cell formation

The graphite based cells used in this work comprised of a stack of dense and mesoporous titanium dioxide (TiO_2_) as electron selective contact, a space layer of mesoporous zirconium dioxide (ZrO_2_), and a counter electrode of graphite flakes. All porous layers were filled with PbI_2_. To form the perovskite layer, the substrate is dipped in a container containing the MAI-solution with the graphite-side facing the solution. Photographs of the cell during perovskite evolution can be found in the Supplementary Information. From the top side, the device can be illuminated through the glass substrate enabling tracking of the measured parameters. Thereby, EQE data and the PL spectra were recorded *in-situ* over a MAI-immersion period of 30 minutes with the analysis providing detailed time-resolved data about device performance and crystallization properties. To ensure reproducibility, the experiments were repeated for each measurement configuration with an overall count exceeding 50 individual cells. The variety of results from different cells as presented in the Supplementary Fig. S6 shows that there are slight deviations in the time constants, giving nevertheless a consistent behavior in the progress of the stages (i to iv) For the sake of clarity, one exemplary curve for each method is presented; additional data can be found in the Supplementary Information.

### Photoluminescence measurement

The photoluminescence (PL) properties were tracked using a fiber coupled PL spectroscopy setup^32^. Locally excited with a frequency doubled Nd:YAG laser (532 nm), applying a photon flux of 2.68 × 10^17^ photons/(s cm^2^) (near to real cell operating conditions), the emitted light is spectrally resolved using a diffraction grating and detected by a silicon charge coupled device (Si-CCD). Since the excitation spot of 2.8 × 10^−2^ cm^2^ (calculated from FWHM) is smaller than the device area, the measurement yields an average value of the illuminated area. All PL measurements were performed without contacting the cell and using a continuous mode with an integration time of 0.1 s and a repetition rate of 10 Hz. The measurements were performed at ambient temperature with no significant temperature increase related to the sample illumination. In the post-annealing stage, the lateral distribution of the PL emission was analyzed by mapping the cell’s active area using confocal microscopy^33^.

### Spectral Response/EQE

The time-resolved EQE (or spectral response) measurements were conducted with a laser-based setup developed in-house at Fraunhofer ISE^33^. The cells were short-circuited using a transimpedance amplifier and illuminated by monochromatic radiation at both 350 and 700 nm simultaneously^34^. In addition, steady-state bias light from LEDs emitting at 465 nm and 660 nm was applied and set to 1.1 × 10^16^ photons/(cm² s), equivalent to approximately 0.1 suns bias irradiance for the completed devices. The monochromatic radiation was frequency-modulated by mechanical chopper wheels at 70 and 90 Hz (for 700 nm and 350 nm, respectively). Lock-in amplifiers were used to distinguish alternating current generated from the monochromatic radiation and direct current resulting from the bias light. Linearity of the devices is assumed so that their differential current response to the chopped monochromatic radiation is approximated as EQE^34^.

The setup has been calibrated by a silicon reference solar cell and fluctuations of the monochromatic light were corrected by using a silicon monitor diode. The illuminated area was significantly larger than the device so that the active cell area was defined by a shadowing mask of approximately 0.4 cm² which was placed on top of the glass.

The datasets generated and analysed during the current study are available from the corresponding author on reasonable request.

## Electronic supplementary material

Supplementary Information
