# Supplementary material for: Distinguishing crystallization stages and their influence on quantum efficiency during perovskite solar cell formation in real-time
Source: Sci Rep. 2017 Nov 2;7:14899. doi: 10.1038/s41598-017-13855-6 (PMC5668251; doi:10.1038/s41598-017-13855-6)
Supplement: Supplementary file 1 — Supplementary Information [file 41598_2017_13855_MOESM1_ESM.doc]

Supplementary Information

**Distinguishing crystallization stages and their influence on quantum efficiency during perovskite solar cell formation in real-time**

Lukas Wagner*, Laura E. Mundt*, Gayathri Mathiazhagan, Markus Mundus, Martin C. Schubert, Simone Mastroianni, Uli Würfel, Andreas Hinsch and Stefan W. Glunz

**Solar cell fabrication**

FTO coated glass plates of dimension 100 x 100 mm were used initially. The TCO was patterned by laser ablation. The plates were cleaned in an ultrasonic bath for 2 minutes at 50 °C in Mucasol detergent and for 1 minute at 50 °C in deionized water. Finally, the plates were rinsed in isopropanol and blow dried with nitrogen. A 25-30 nm thick compact TiO_2_ was deposited by spray pyrolysis on the cleaned glasses. For this purpose, a solution containing 0.05M of titanium disisopropoxy (bis) acetyl acetone (Sigma Aldrich) diluted in ethanol (>= 99.5% from Carl Roth) was used. A layer of approximately 600 nm of mesoporous TiO_2_ was then screen printed with a paste prepared by mixing Dyesol DSL‑18NRT with terpineol in 1:0.5 weight ratio. The film was sintered at 500 °C for 30 min. Approximately 800 nm of porous ZrO_2_ space layer was screen printed (Solaronix Zr-Nanoxide ZT/SP) and sintered at 500 °C for 30 min. The stack was finished by screen-printing an approximately 7 µm thick graphite counter electrode on top (Solaronix Elcocarb B/SP). This stack resulted in an active area of 0.4 cm². The plates were finally sintered at 400 °C for 30 min. The plates were then cut to substrates of 25 x 25 mm dimensions and copper cables were soldered on the corresponding TCO patches. Electrical contact between the graphite patch and the wires was established with silver paste (G3692 Acheson silver DAG 1415). The cells were transported inside a nitrogen-filled glove box where they were heated to 120 °C for 15 minutes to remove moisture. The PbI_2_ layer was formed by dropping 7 µl of a 1.2M solution of PbI_2_ (TCI, 99.99%) in N,N-dimethylformamide (DMF) (Sigma-Aldrich, 99.8%) at 70 °C close to the active area. After waiting for 1 minute, the film was spin cast 2500 RPM for 10 seconds and annealed at 70 °C for 30 minutes. The MAI solution for dipping consisted of 0.062M of methyl ammonium iodide (Dyesol) in dried isopropanol (Merck, >=99.9%).

All measurements were performed at room temperature and in ambient air.

**Optical appearance during MAI-reaction**

Figure S1 a-g show photographs taken during the transformation of a PbI_2_-filled porous structure while dipped in MAI-solution. One can see that the active area is initially yellow colored by the PbI_2_. The region without carbon layer (outside the active area) turns dark quickly upon MAI-immersion, indicating formation of MAPbI_3_. Within the active area, the transformation to perovskite proceeds slower, most probably due to a retardation of MAI infiltration by the graphite capping layer. Remarkably, the transformation occurs not from the sides to the center but rather homogeneously throughout the active area. Following the RGB signal intensity evolution averaged over the active area with time (Figure S1 h) it can be noted that it saturates after approximately 9 min.

**
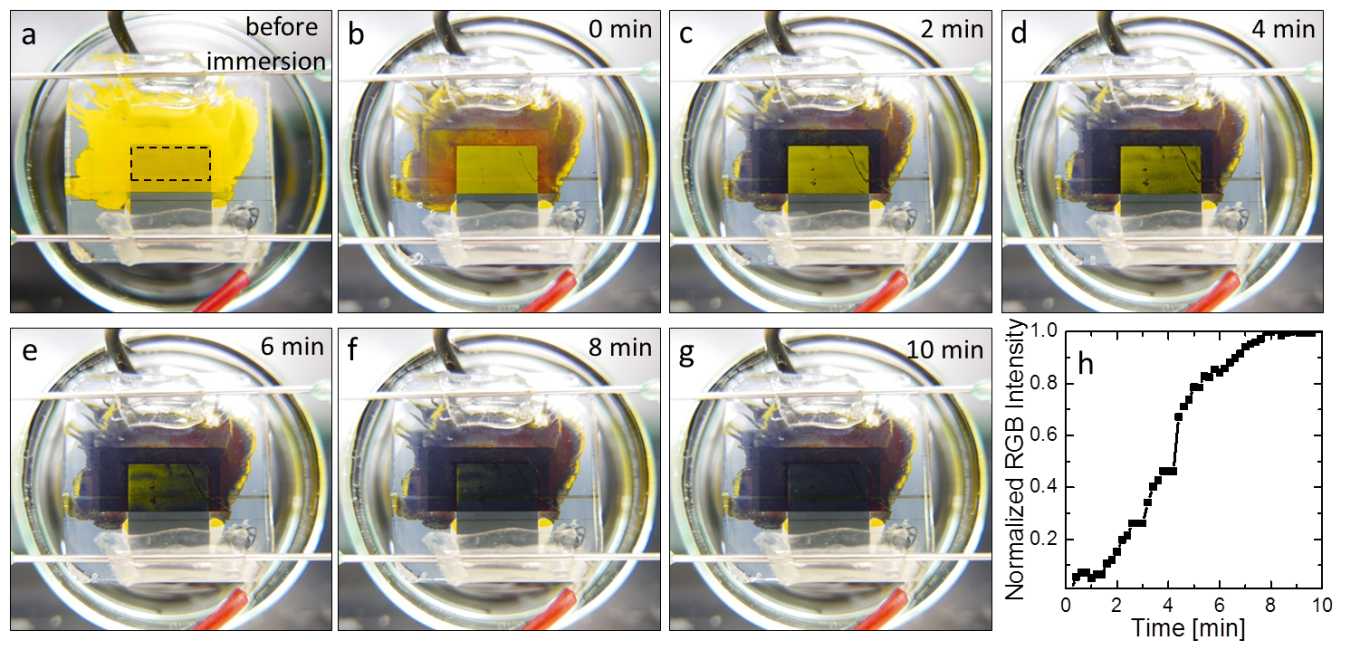
**

**Figure S1 |** Photos of the PbI_2_-filled substrate seen through the glass side before a) and directly (< 1 s) after MAI-immersion b), as well as during dipping at 2 min c), 4 min d), 6 min e), 8 min f) and 10 min g). The active area of the cell is sketched by the dashed rectangle in photo (0.4 cm^2^) a). Plot h) shows the normalized RGB signal intensity evolution with time in the active area, effectively representing the light absorption.

**Photoluminescence measurements**

For two cells we show the complete PL emission properties during MAI-immersion in Figure S2-S4. Small time shifts can occur since these measurements were performed on individual cells and the exact time of the MAI solution injection may vary slightly. The PL emission spectrum was fitted by a Gaussian function:
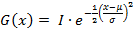


**Figure S2 |** PL intensity during MAI-immersion. (*I*)

**Figure S3 |** PL peak position during MAI-immersion. (
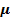
)

**Figure S4 |** PL peak width during MAI-immersion. (
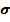
)


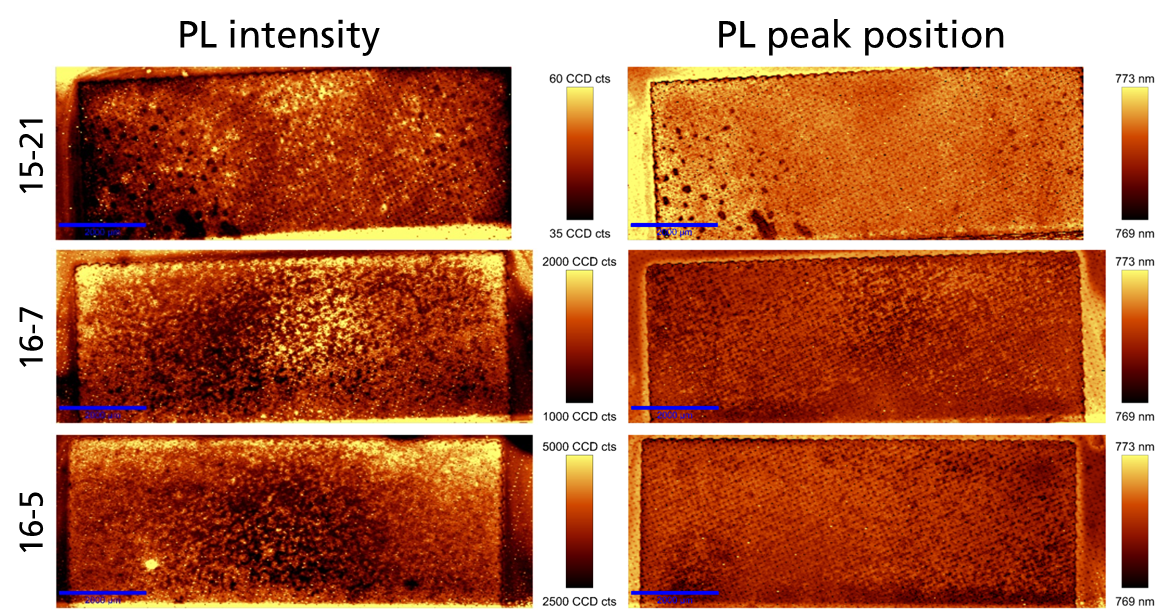


**Figure S5 |** Spatially resolved PL intensity obtained by a confocal PL microscope. Measurements were performed after drying and annealing of the film. The darker spots which can be observed at the left and right of the two images are due to the effect/influence of the PbI_2_ solution dropped onto the graphite layer. The blue scale bar corresponds to 2 mm.

**Spectral response / EQE measurements**

Figure S6 shows EQE measurements that were taken parallel under 350 and 700 nm illumination for three different cells. Besides the behavior upon MAI immersion, also the measurements for a “drying” and post-annealing” condition are shown. For “drying”, the MAI-filled container was replaced by an empty one and the cell was monitored for 30 minutes of drying during which the residual isopropanol evaporates. Finally, the cell was annealed at 70 °C on a hotplate for 30 min, after which the “post-annealing” curve was recorded. Figure S6 shows that especially after annealing, there is a significant increase in EQE. It is beyond the scope of the present paper to analyze the effects during these processing steps.


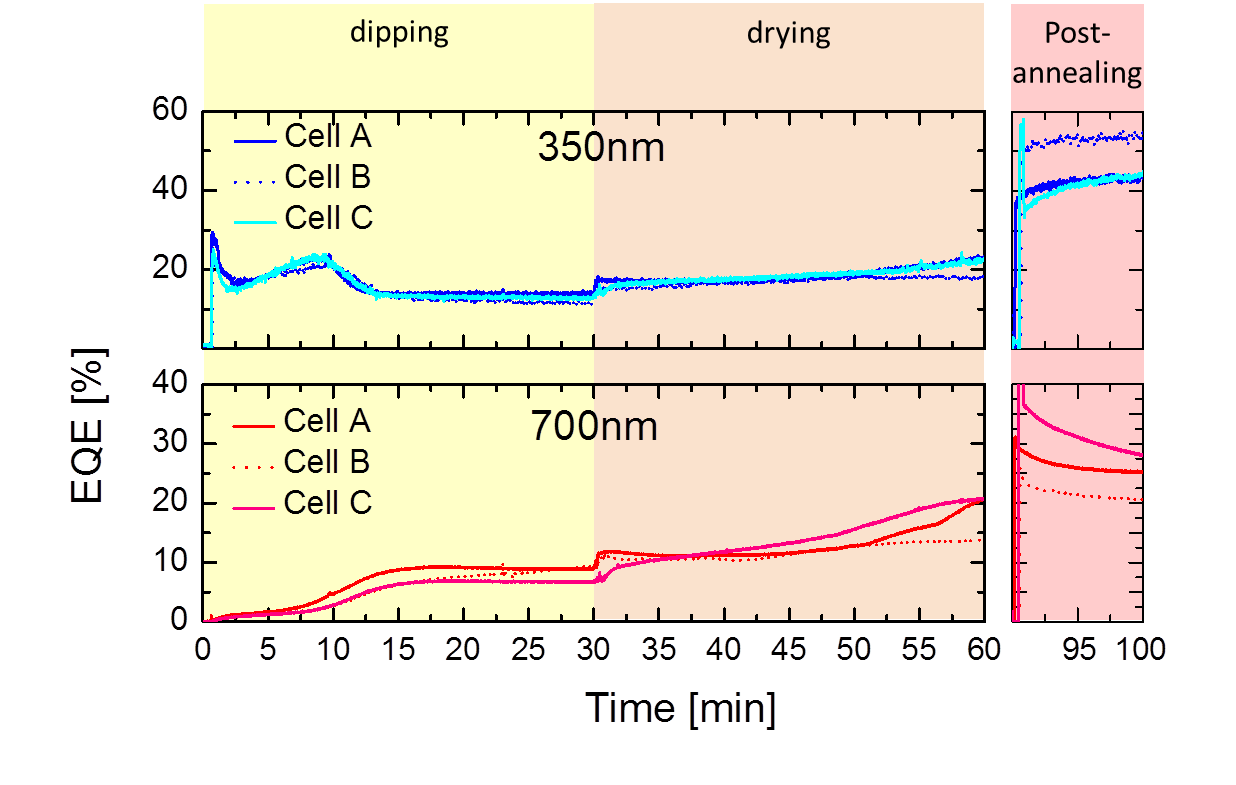


**Figure S6 |** External quantum efficiency, simultaneously tracked at 350 nm and 700 nm for three cells.

**I-V Measurements under white light illumination - Electrochemical effects**

Photocurrent measurements were also obtained under white light illumination. However, here the direct effect of the crystallization was superimposed by effects that we attribute to electrochemical processes induced from the blue part of the illumination source.

I-V measurements were recorded with a potentiostat (ivium CompactStat.h 10800). The devices were kept in short circuit (I_SC_) throughout the measurement, except for the annealing step. For white light illumination, a 15 x 15 cm^2^ array of white LED was used (ivium iviSUN). The spectrum of the LEDs is displayed in Figure S7. The cells were measured under the LED array set to 14 klx intensity at a distance of approximately 5 cm, corresponding to roughly 0.1 suns.

**
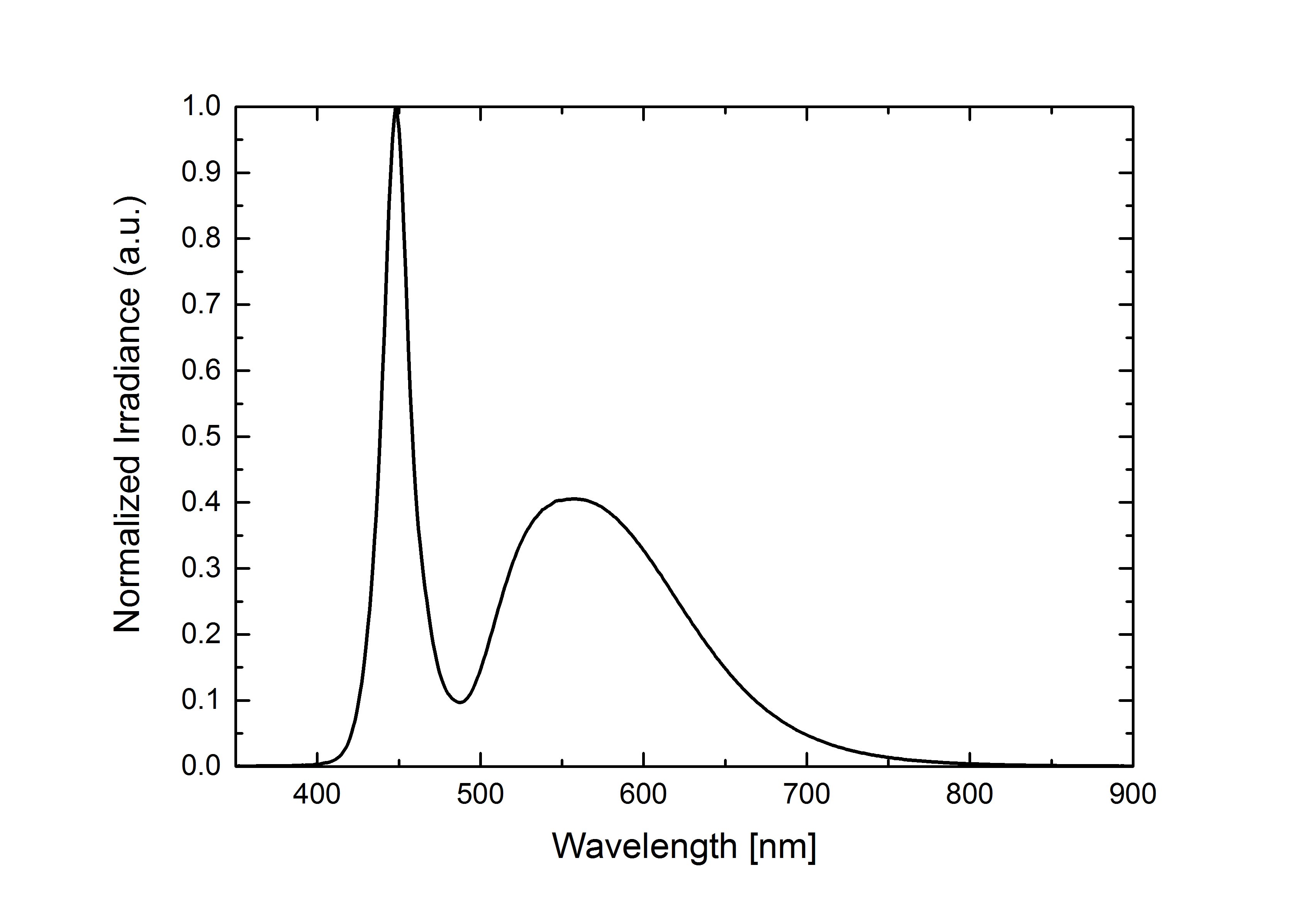
**

**Figure S7 |** Measured emission-spectrum of the white light LED array.

Interestingly, a substantial electrical current was observed already from the very beginning of the measurement, i.e. while the cell still appeared yellow from the dominant presence of PbI_2_. The measured current followed a reproducible wave-like pattern within the first 10 minutes before reaching a stable level for the rest of the dipping period (cf. Figure S8). This pattern was most dominantly observed in the EQE measurement at 350 nm (cf. Figure S6). Moreover, the drying and post-annealing as carried out for Figure S6 is presented.

Remarkably, the transparent MAI-solution had turned yellow while the cell was dipped for 30 minutes under illumination, which we ascribe to the formation of tri-iodine (I_3_^−^). We attribute the initial presence of an electric current and the wave-like pattern to electrochemical processes caused by short wavelength absorption in the TiO_2_ and in the PbI_2_. To verify this, “unfilled” mesoscopic cells of m-TiO_2_/m-ZrO_2_/graphite (i.e. without PbI_2_) were simultaneously illuminated with monochromatic light at 350 and 700 nm while being immersed in the MAI-solution. A photocurrent was only observed under the 350 nm light (cf. Figure S9). As the TiO_2_ absorbs at 350 nm, while being transparent at 700 nm, photo-excited holes are generated upon illumination at 350 nm by direct band-gap excitation. We expect that like in the case of dye solar cells (DSCs) the holes from the photo-excited dye are then taken up by iodide from the MAI solution in a two-electron redox reaction (3I^-^ 🡪 I_3_^−^ + 2e^-^) and diffuse to the graphite counter electrode. In order to further analyze this behavior, in an additional experiment we immersed now a PbI_2_-filled cell in a DSC-type iodide-containing electrolyte (1-methyl-3-propylimidazolium iodide mixed with iodine in isopropanol). The electrolyte was chosen such that no perovskite formation was induced upon immersion. We observed a sensitized electric current for illumination under excitation wavelengths of 350 and 500 nm but not for wavelengths below the band-gap of PbI_2_ of 550 and 700 nm as expected. Also, the photocurrent under 350 nm dropped quickly while the current at 500 nm prevailed (cf. Figure S10). As PbI_2_ absorbs from the UV until approximately 520 nm, this points in the direction that also here PbI_2_ is acting as an efficient sensitizer on TiO_2_.


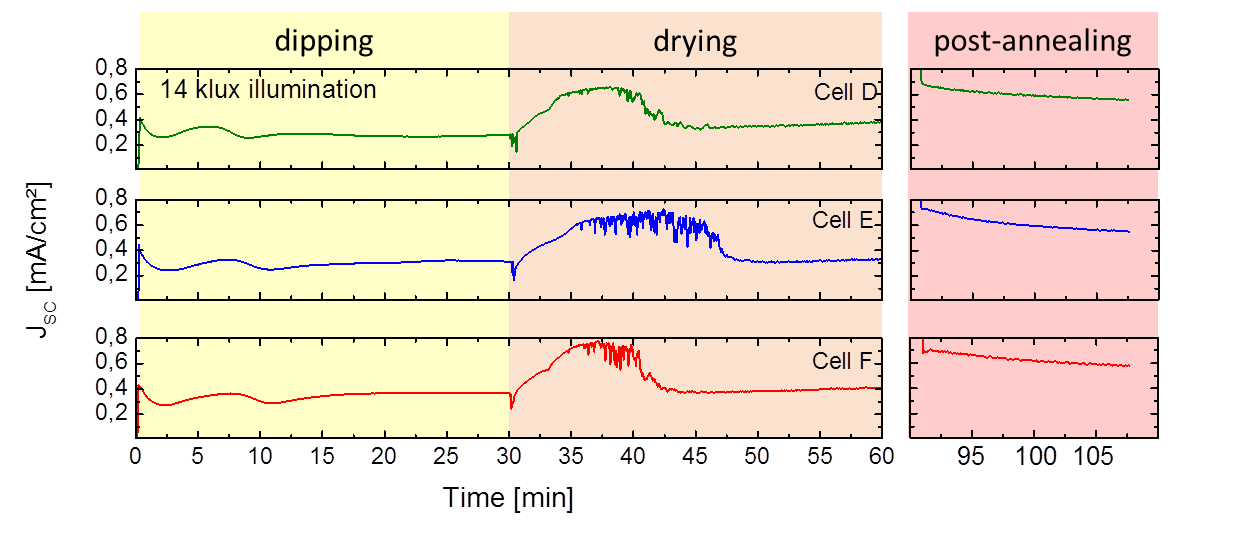


**Figure S8 |** Short circuit current density J_SC_ during MAI-immersion for three cells under 14 klux white LED illumination (roughly 0.1 suns).


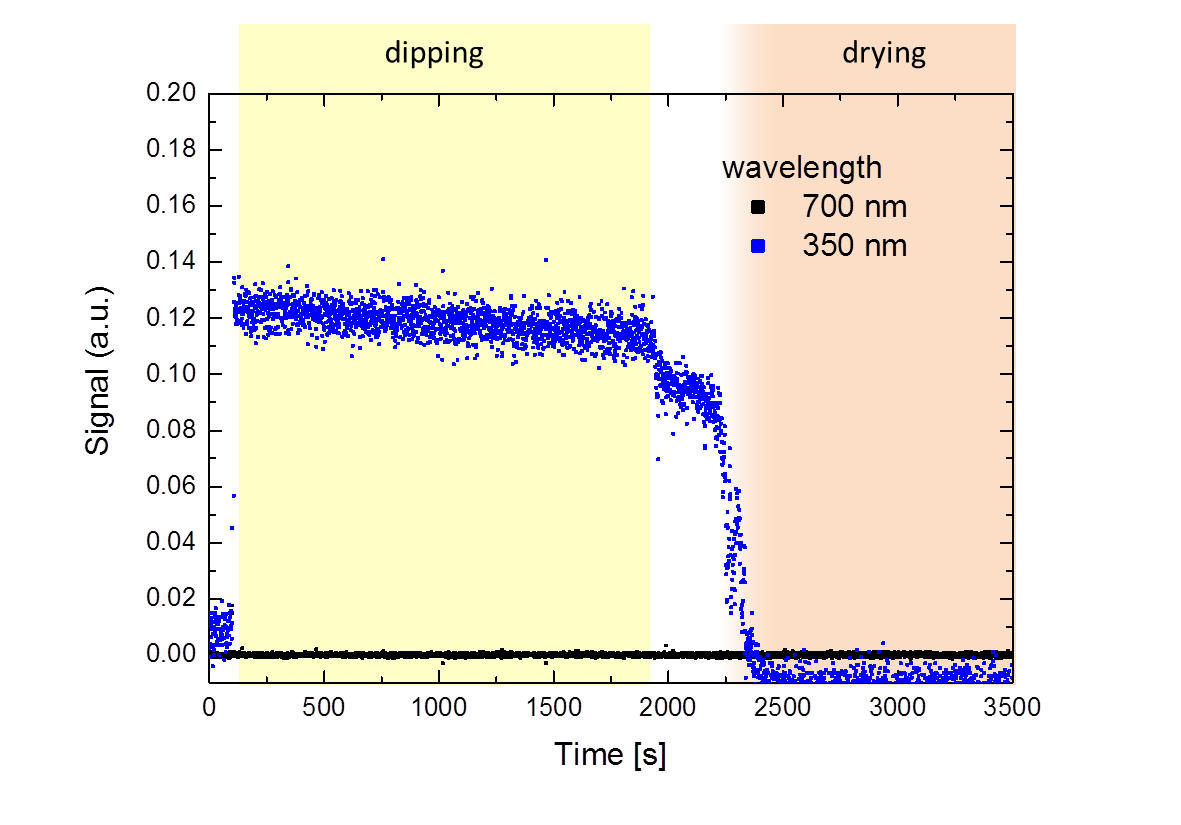


**Figure S9 |** Simultaneously tracked current (in arbitrary units) from 350 nm and 700 nm monochromatic radiation, for MAI-immersion and consecutive drying of the film in air of an „unfilled” cell, i.e. a cell without PbI_2_ layer.


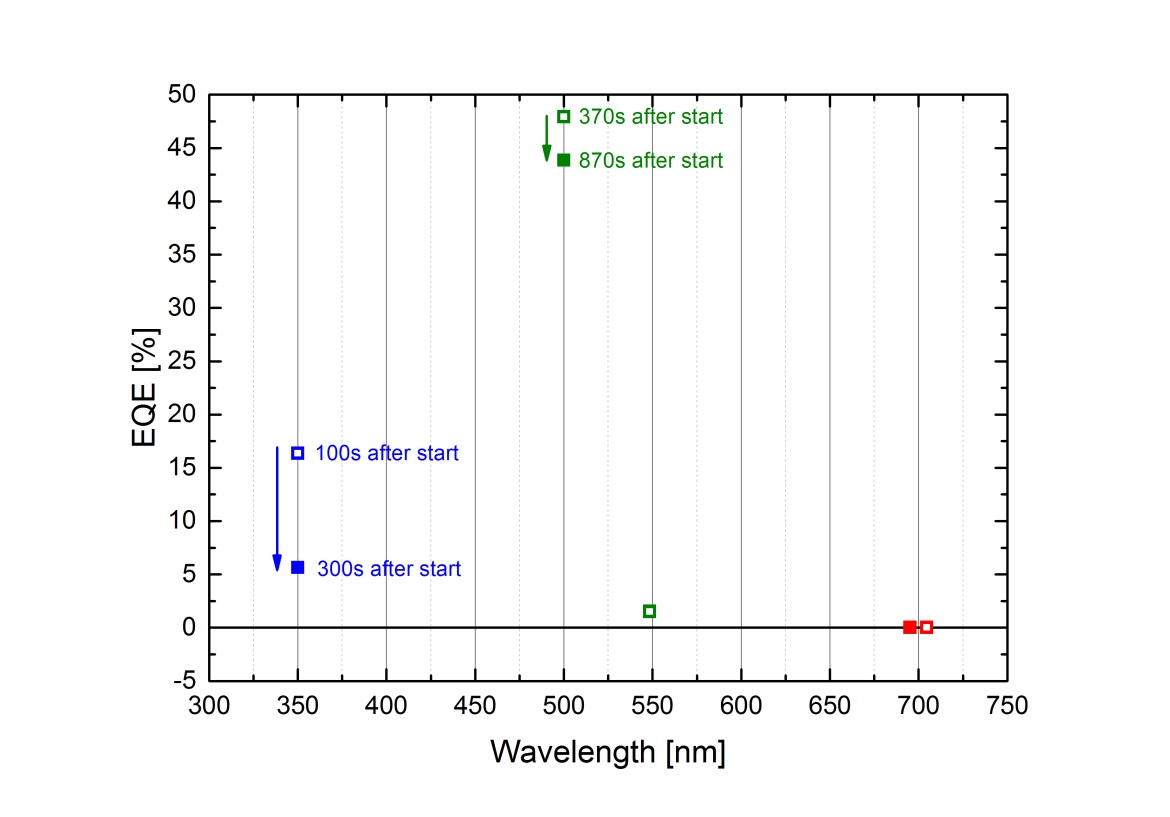


**Figure S10 |** External quantum efficiencies at 350, 500, 550, and 700 nm of a fully processed perovskite cell, immersed in an iodine-rich electrolyte.

**Analysis of devices with intermitted** **crystallization**

In addition to the real-time crystallization analyses, a set of devices was produced where the perovskite crystallization was interrupted during the immersion of cells into the MAI solution after 3, 8, 15 and 30 minutes. The reaction times were chosen to reflect the end of each reaction stage. We note that contrarily to sequentially deposited perovskite solar cell architectures where, after the perovskite formation, hole/electron transport layer deposition and back contact metallization evaporation under low pressure are subsequently carried out, in graphite based devices such an interrupted crystallization approach provides a more direct and prompt insight on the crystallization stage. However, this approach is still prone to include post-crystallization artefacts which are excluded in the real-time approach.

To interrupt the crystallization process, PbI_2_-filled graphite substrates were first immersed for a defined time in a solution of MAI dissolved in dry isopropanol. The reaction was then stopped by dipping the substrate in pure isopropanol. Hereby, it was assumed that the excess of unreacted MAI is quickly depleted and the reaction from PbI_2_ to MAPbI_3_ terminates.

**X-ray diffraction (XRD) measurements**

XRD measurements were carried out on an X’pert Pro x-ray diffraction system (PANalytical) with a ceramic tube Cu-anode x-ray source (λ = 1.54184 Å) operated in grazing incidence geometry.

For this characterization, the graphite based cells had to be prepared in a special way: while a free-standing PbI_2_ film reacts within few seconds to MAPbI_3_ when immersed in MAI solution, the reaction is strongly retarded by the diffusion of the MAI ions through the porous graphite layer in the active area (cf. Fig. S1). For XRD analysis, a set of cells was prepared such that only the reaction stage of the absorber material and in the photoactive layer was detected. Therefore, after spin-coating, the excess PbI_2_ layer was wiped away with a tissue wetted with DMF over the entire substrate except for the active area. After the reaction with MAI, the graphite electrode was removed with an adhesive tape to further ensure that no XRD signal originating from inactive perovskite remains in the graphite is detected and only the reaction stage of the perovskite inside the photoactive layer, where it is embedded in the porous TiO_2_ and ZrO_2_, was characterized

Figure S11 shows the XRD spectra of four such samples immersed in MAI for 3, 8, 15 and 30 minutes, respectively. For the analysis of the formation of perovskite from the PbI_2_ layer, the reflection peaks at 2θ=14.0° for the perovskite and 2θ=12.6° for the PbI_2_ can representatively be observed. As also listed in Table S1, the net heights of the peaks of PbI_2_ are reduced in comparison to pristine PbI_2_ as the cell is dipped in MAI. Still, reflection spectra characteristic for PbI_2_ remain for all samples. The evolution of the perovskite can be clearer seen from the net heights of the peaks of perovskite. This peak is still very low for dipping times of 3 minutes and rises by a factor of 10 for the sample dipped for 8 minutes. For this and the following dipping times, it remains at approximately the same height. We note that the height of the reflection does not give direct information on the amount of material present.

From the full width half maximum (FWHM) of the perovskite peak, the evolution of the grain sizes can also be estimated. From Table S1 it can be seen that the FWHM has the highest value for the 3 minute device. It is similar for the devices of 8, 15, and 30 minutes MAI immersion. According to the Scherrer equation, the grain size is reciprocally proportional to the FWHM. This means that the grains grow until an immersion time of 8 minutes, from where on the size remains stable. This observation is in good agreement with the findings from the peak red-shift shift in the PL characterization in Figure 2b.

It is not straight-forward to apply the Scherrer equation to perovskite crystals growing inside porous media. Yet, assuming a shape factor of 1, this equation yields a grain size of approximately 20 nm for the stabilized FWHMs (8 minutes and longer). This is in the same range as the pore particle size of which the porous TiO_2_ and ZrO_2_ layers are comprised.


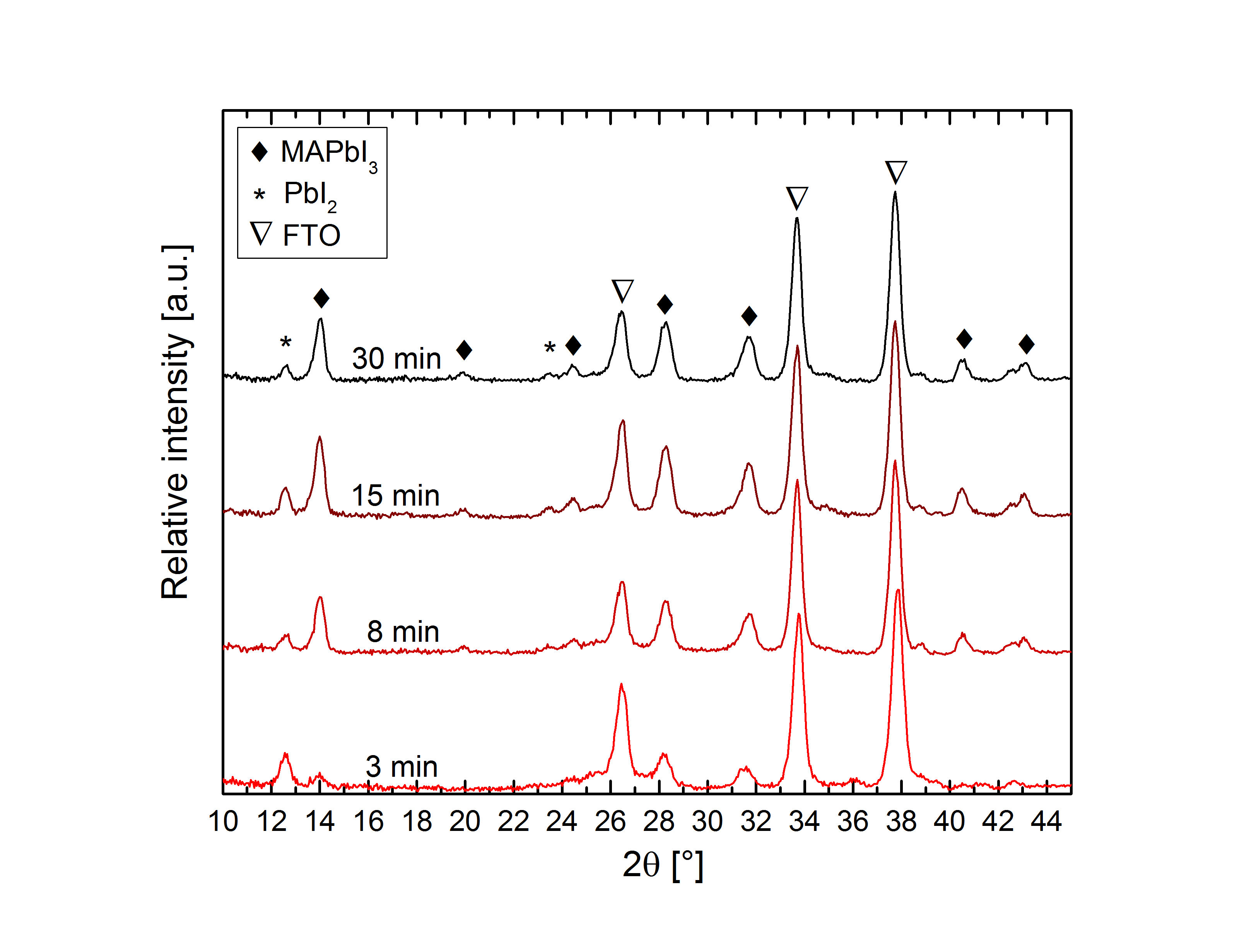


**Figure S11 |** XRD spectra of perovskite cells immersed in MAI solution for 3, 8, 15, and 30 minutes, respectively. Indices correspond to reflexes perovskite (♦), PbI_2_ (*), and the FTO substrate (∇).

| **Dipping time** | **PbI_2_ net height [a.u.]** | **MAPbI_3_ net height [a.u.]** | **MAPbI_3_ FWHM [°]** |
| --- | --- | --- | --- |
| **0** | 1607.9 | n.a. | n.a. |
| **3** | 1041.0 | 257.4 | 0.565 |
| **8** | 565.8 | 2167.6 | 0.449 |
| **15** | 959.8 | 2730.8 | 0.465 |
| **30** | 525.3 | 2428.6 | 0.434 |

**Table S1.** Details of the XRD spectra of graphite cells immersed in MAI for 0, 3, 8, 15, and 30 minutes, as shown in Figure S11. Displayed are the net heights of the first reflection peaks of PbI_2_ (at 2θ=12.6°) and MAPbI_3_ (at 2θ=14.0°) as well as the full width half maximum (FWHM) of the respective peak of MAPbI_3_.

**UV vis transmittance spectroscopy**

UV-Vis transmittance measurement where performed on a Perkin Elmer Lambda 950 UV-Vis-NIR spectrometer. The same samples used for the XRD measurements were characterized. To ensure that only light passing through the cell’s active area was detected, the entrance slit of the integrating sphere was patterned with a photomask. Still, a significant offset between the transmittance signals of the samples is present. The transmittance measurement is very sensitive to variations in the thickness of the observed layer as well as contaminations by unwanted materials. Therefore, we attribute the differences mainly to variations in layer thickness and residues of the graphite top electrode, arising from the subsequent removal of the graphite layer. For the samples where the perovskite layer was readily formed, it can be presumed that the perovskite adheres more strongly to the graphite in comparison to PbI_2_ resulting in a thin graphite capping layer for these samples.

In Figure S12 it can clearly be seen that after 8 minutes, the perovskite film has formed as the transmittance is strongly decreasing for wavelengths smaller than the band gap op MAPbI_3_ of around 780 nm. The presence of PbI_2_ in the pristine (“0 min”) sample is indicated by the band-gap of around 500 nm. In accordance to the results of the XRD characterization, the transmittance of the “3 minute” sample resembles mainly the one of pristine PbI_2_, although a slight dip in transmittance around the band-gap of MAPbI_3_ can be observed. These observations are as well in good agreement with the PL characterization from Figure 2.


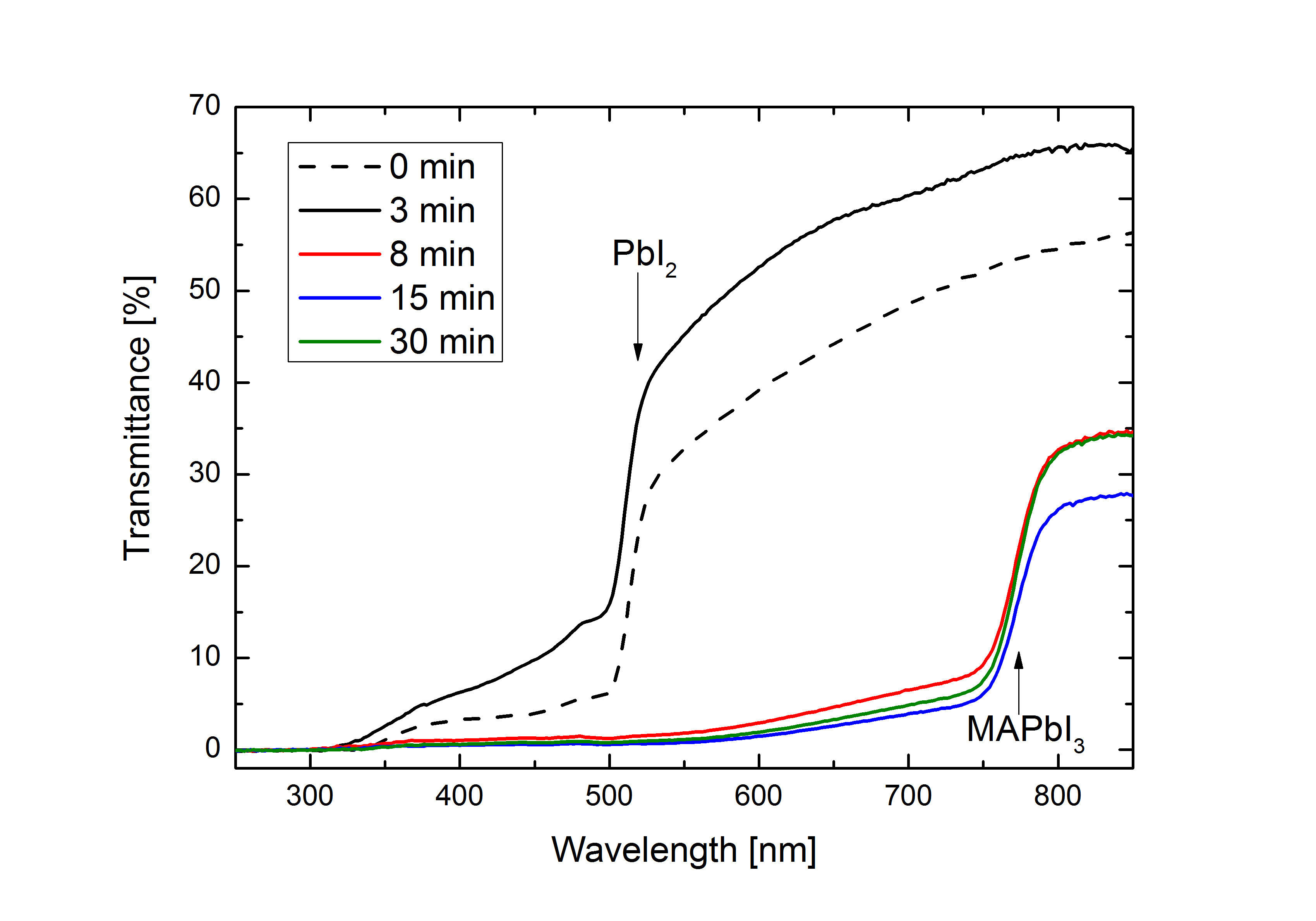


**Figure S12 |** UV-Vis transmittance spectra of cells with removed graphite layer for different MAI-immersion times. The vertical arrows indicate the band-gaps of PbI_2_ and MAPbBI_3_.

**I-V characteristics of cells with interrupted MAI-dipping**

To study the I-V characteristics of perovskite solar cells for different MAI-immersion times PbI_2_ filled two samples of graphite cells each were dipped in MAI solution for 3, 8, 15 and 30 minutes. The immersion was interrupted by dipping the cells in dry isopropanol. The cells were characterized under a class A LED solar simulator. We note that due to changes in the screen-printing procedure, a slightly varied cell structure in comparison to the samples presented in the other experiments had to be used.

Figure S13 shows the reverse scan IV-characteristics of the respective cells. Revealingly, while there is some statistical deviation among the cells, the curves clearly form two groups: cells immersed for 8 minutes and longer show all similar I-V curves. The I-V curves of samples immersed for 3 minutes only, however, display a lower J_SC_ and a higher V_OC_.

The lower J_SC_ for the “3 min” samples can easily be explained by the lower amount of perovskite present in the cells, by which less light is absorbed and converted to a photocurrent (cf. Figure S12). The V_OC_ on the other hand is remarkably high for a graphite based cell (1046.0 mV for sample 2). In accordance with the observation that the PL intensity is at its maximum for a 3 minute dipping time (cf. Figure 2), this supports the interpretation that non-radiative recombination is very low at this stage of perovskite formation. The most efficient device (sample 3 in Figure S13) yielded a V_OC_ of 910.8 mV, a J_SC_ 17.3 of mA/cm^2^, and a fill factor of 55.5 %, resulting in a power conversion efficiency (PCE) of 8.8 % for the reverse scan (active area 0.4 cm^2^). During maximum power point tracking, the PCE remained stable around 8.3 % for a measurement duration of 60 seconds. Although the real-time characterization showed that the EQE evolves further after 8 minutes upon MAI immersion, in the interrupted experiment where the cells are taken out of the solution and dried, a stabilized photocurrent is measured already after this time of immersion.


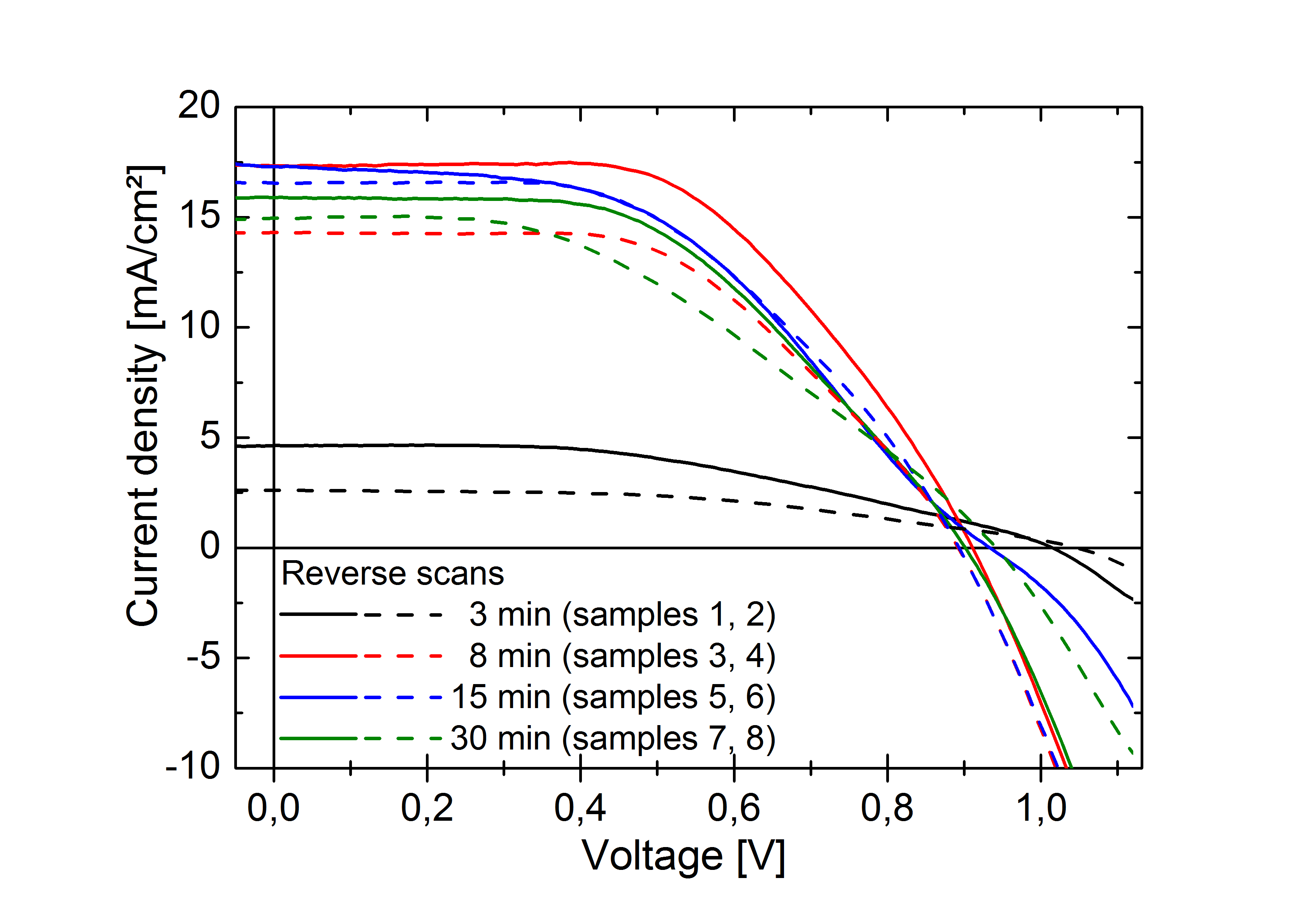


**Figure S13 |** Reverse scan I-V curves of 2-step processed perovskite solar cells for MAI immersion times of 3, 8, 15, and 30 minutes.
